# SMA-MAP: A Plasma Protein Panel for Spinal Muscular Atrophy

**DOI:** 10.1371/journal.pone.0060113

**Published:** 2013-04-02

**Authors:** Dione T. Kobayashi, Jing Shi, Laurie Stephen, Karri L. Ballard, Ruth Dewey, James Mapes, Brett Chung, Kathleen McCarthy, Kathryn J. Swoboda, Thomas O. Crawford, Rebecca Li, Thomas Plasterer, Cynthia Joyce, the Biomarkers for Spinal Muscular Atrophy Study Group, Wendy K. Chung, Petra Kaufmann, Basil T. Darras, Richard S. Finkel, Douglas M. Sproule, William B. Martens, Michael P. McDermott, Darryl C. De Vivo, the Pediatric Neuromuscular Clinical Research Network, Michael G. Walker, Karen S. Chen

**Affiliations:** 1 Spinal Muscular Atrophy Foundation, New York, New York, United States of America; 2 Walker Bioscience, Carlsbad, California, United States of America; 3 Myriad RBM, Austin, Texas, United States of America; 4 Departments of Neurology and Pediatrics, University of Utah School of Medicine, Salt Lake City, Utah, United States of America; 5 Departments of Neurology and Pediatrics, Johns Hopkins University, Baltimore, Maryland, United States of America; 6 New England Research Institutes, Watertown, Massachusetts, United States of America; 7 BG Medicine, Waltham, Massachusetts, United States of America; 8 The Pediatric Neuromuscular Clinical Research Network, New York, New York, United States of America; University of Edinburgh, United Kingdom

## Abstract

**Objectives:**

Spinal Muscular Atrophy (SMA) presents challenges in (i) monitoring disease activity and predicting progression, (ii) designing trials that allow rapid assessment of candidate therapies, and (iii) understanding molecular causes and consequences of the disease. Validated biomarkers of SMA motor and non-motor function would offer utility in addressing these challenges. Our objectives were (i) to discover additional markers from the Biomarkers for SMA (BforSMA) study using an immunoassay platform, and (ii) to validate the putative biomarkers in an independent cohort of SMA patients collected from a multi-site natural history study (NHS).

**Methods:**

BforSMA study plasma samples (N = 129) were analyzed by immunoassay to identify new analytes correlating to SMA motor function. These immunoassays included the strongest candidate biomarkers identified previously by chromatography. We selected 35 biomarkers to validate in an independent cohort SMA type 1, 2, and 3 samples (N = 158) from an SMA NHS. The putative biomarkers were tested for association to multiple motor scales and to pulmonary function, neurophysiology, strength, and quality of life measures. We implemented a Tobit model to predict SMA motor function scores.

**Results:**

12 of the 35 putative SMA biomarkers were significantly associated (p<0.05) with motor function, with a 13^th^ analyte being nearly significant. Several other analytes associated with non-motor SMA outcome measures. From these 35 biomarkers, 27 analytes were selected for inclusion in a commercial panel (SMA-MAP) for association with motor and other functional measures.

**Conclusions:**

Discovery and validation using independent cohorts yielded a set of SMA biomarkers significantly associated with motor function and other measures of SMA disease activity. A commercial SMA-MAP biomarker panel was generated for further testing in other SMA collections and interventional trials. Future work includes evaluating the panel in other neuromuscular diseases, for pharmacodynamic responsiveness to experimental SMA therapies, and for predicting functional changes over time in SMA patients.

## Introduction

Spinal Muscular Atrophy (SMA) is a rare genetic neuromuscular disease caused by the loss of the Survival Motor Neuron 1 gene (SMN1). The depletion of the SMN protein in cells causes death of alpha motor neurons, resulting in extreme weakness in proximal muscles, particularly those required for breathing and posture. The disease largely manifests in children with a continuum of severity and developmental onset in which the most severely affected (type 1) have symptoms before 6 months of age and are unable to sit independently and often die within a few years of birth, moderate disease patients (type 2) have symptoms by 18 months and are unable to walk independently, and patients with milder forms (type 3) have onset after 18 months and are able to walk but may lose the capacity to ambulate over time. SMA is the epitome of a disease with high unmet medical need, as 1) there is no effective treatment, 2) the most severely affected patients succumb to respiratory failure, and 3) all patients experience significant progressive functional decline and morbidity due to extreme muscle weakness and atrophy.

However, there has been much progress in the development of new SMA therapeutics and in the understanding of the biology of the disease and SMN. New drugs being expressly developed for SMA and similar diseases include ISIS-SMNRx (Isis Pharmaceuticals), Olesoxime (Trophos), and RG3039 (Repligen), with a number of other programs in preclinical development [1]. As new drugs advance through the clinic, outcome measures and biomarkers will be utilized and validated by the SMA research community. Several clinical studies using existing nervous system or other drugs have been conducted in SMA including albuterol, gabapentin, phenyl butyrate, riluzole, and valproic acid [2]–[9]. While none of the drugs have yet produced robust positive effects in larger or well-controlled clinical trials, the field gained critical expertise in the execution of trials, testing of study designs, coordinating clinical networks and building and validating outcome measures and also biomarkers. Several motor function scales (including SMA-specific measures like the Hammersmith Motor Function Scale or HFMS), quality of life scales (PedsQL neuromuscular module, respiratory measures, strength tests, and several putative biomarkers for SMN transcript and protein as well as other outcome measures have already been piloted in these intervention studies and in natural history studies and are ready for use and validation in new drug trials [10]–[22].

However, new SMA biomarker investigation is an emerging area of research, and prior efforts included exploring volumetric MRI imaging and electrical impedance myography [23], [24]. Development of non-SMN molecular biomarkers remains an area for opportunity for SMA, and the recent BforSMA study was a major advance in the discovery of new biomarkers for this disease [25], [26].

A biomarker panel that regresses to motor function scales likes the HFMS, MHFMS, or HFMSE has several possible uses in pre-clinical and clinical studies. Performing the motor score assessment causes fatigue in the patient; differences in effort and differences in the encouragement given the patient by the assessor cause variation in the motor score unrelated to changes in clinical status. A biomarker panel may be a more reproducible measure of disease status than the actual motor score, and may reduce the fatigue and discomfort in the patient, and be less vulnerable to inadvertent unblinding. By providing a more reproducible measure of clinical status, the biomarker panel may provide more reproducible measures of response to drug, potentially decreasing sample size and duration of trials. The biomarkers found in the human studies have analogs in animals, and these may be useful pre-clinical studies and animal models of SMA.

Here we describe the discovery and validation of candidate SMA blood biomarkers using both chromatographic and immunoassays, in two different SMA patient populations from the BforSMA study and an SMA natural history study, which produced a validated 27 analyte panel (SMA-MAP) [15]. Unless otherwise stated, the analyses included types 1, 2, and 3 patients.

## Results

### Discovery Phase: BforSMA

**Figure 1:**
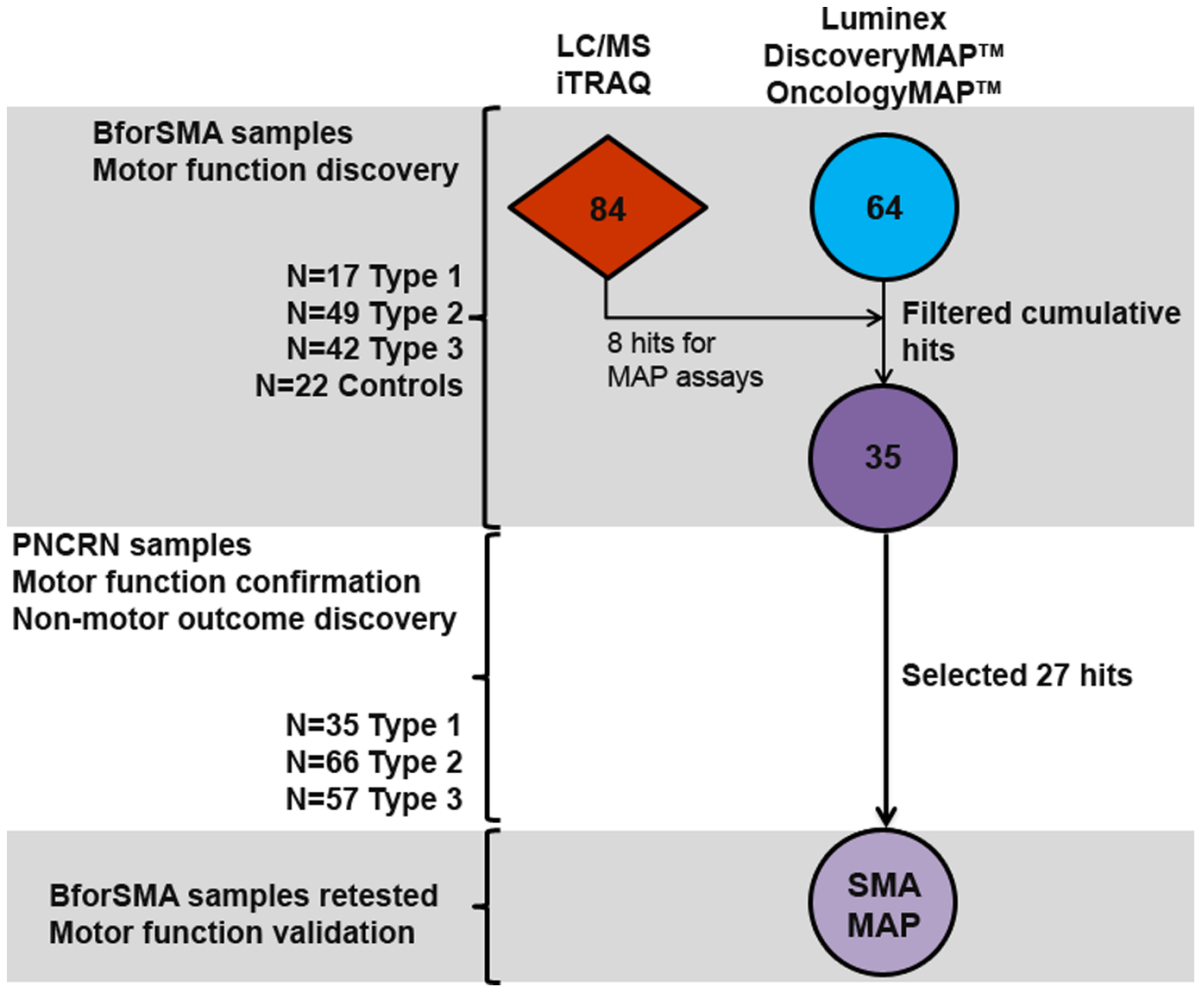
SMA plasma biomarker discovery campaign and confirmation schematic. Analyte markers were identified in different discovery campaigns in two platforms. BforSMA samples were screened in LC/MS using iTRAQ technology, generating 84 markers that regressed with SMA motor function (MHFMS). Samples from the same study were screened in commercially available Luminex panels, yielding an additional 64 markers that regressed to motor function. There were 14 markers in the MAP panels that were hits in the LC/MS campaign, and 11 of these were repeat hits. New Luminex assays were created to represent the top 8 analytes from the LC/MS analysis. Filtering was performed by evaluation of statistical strength and assay performance, and 35 top analytes were selected for further MAP testing in a new sample set from the PNCRN natural history study. An additional 91 analytes were present in the panels for testing, allowing discovery based on non-motor outcome data that was collected in the PNCRN study. 13 analytes were repeat motor regressors, while 15 were new non-motor analytes. A total of 27 analytes were selected for inclusion to the final SMA-MAP panel, which was validated for reproducibility using unthawed samples from BforSMA.

The overall flow of SMA plasma protein biomarker candidates from the discovery phase through validation and their inclusion in the final SMA-MAP is depicted in Figure 1. The discovery phase yielded 35 putative SMA biomarkers selected to progress into the validation phase. We first re-examined the plasma proteomic data from the BforSMA study, to identify the best proteins for building new immunoassays [26]. Specifically, previously published data on the intensity ratios for the protein analytes (available at neuinfo.org/smabiomarkers and derived from multidimensional liquid chromatography combined with isobaric tag for relative and absolute quantitation or iTRAQ, Table S1) were analyzed against MHFMS for each individual subject using univariate regression [26]. We replicated the initial mathematical analysis excluding non-SMA subjects, and found 84 markers associated with MHFMS (Table 1), with considerable overlap to the prior analysis that identified 97 analytes. A notable difference was the loss of SPP1 as a top motor score regressor in the second analysis. New Luminex assays were created for 8 candidate biomarkers that were among the strongest motor regressors with available reagents: CD93, CDH13, COMP, DPP4, LUM, PEPD, THBS4, and TNXB.

**Table 1:** SMA plasma protein marker that regress to motor function (MHFMS).

BforSMA LC/MS Markers		Correlation to MHFMS	
Protein	Name	R-value	p-value
CILP2	Cartilage intermediate layer protein 2	0.76	<0.001
TNXB	Tenascin XB	0.72	<0.001
CLEC3B	Ctype lectin domain family 3, member B (tetranectin)	0.65	<0.001
TNXB	Tenascin XB	0.60	<0.001
ADAMTSL4	ADAMTSlike 4	0.56	<0.001
THBS4	Thrombospondin 4	0.52	<0.001
COMP	Cartilage oligomeric matrix protein	0.52	<0.001
CRTAC1	Cartilage acidic protein 1	0.48	<0.001
F13B	Coagulation factor XIII, B polypeptide	0.46	<0.001
PEPD	Peptidase D	0.44	<0.001
LUM	Lumican	0.43	<0.001
CD93	Complement component 1, q subcomponent, receptor 1	0.42	<0.001
	Mixed complement C2/B	−0.41	<0.001
APCS	Amyloid P component, serum	−0.39	<0.001
VTN	Vitronectin	−0.38	<0.001
DPP4	Dipeptidylpeptidase 4 (CD26, adenosine deaminase complexing protein 2)	0.38	<0.001
CRP	C-reactive protein, pentraxinrelated	−0.37	<0.001
HBB	Hemoglobin beta	−0.37	<0.001
GSN	Gelsolin	0.37	<0.001
NCAM1	Neural cell adhesion molecule 1	0.35	<0.001
CFI	I factor (complement)	−0.35	<0.001
APOA4	Apolipoprotein AIV	0.35	<0.001
VTN	Vitronectin	−0.35	<0.001
F13A1	Coagulation factor XIII, A1 polypeptide	0.35	<0.001
INHBC	Inhibin, beta C	−0.34	<0.001
RPS27A	Ubiquitin and ribosomal protein S27a precursor	−0.33	0.001
CDH13	Cadherin 13, Hcadherin (heart)	0.33	0.001
	mixed Complement C2/B	−0.33	0.001
C2	Complement component 2	−0.33	0.001
CP	Ceruloplasmin (ferroxidase)	−0.32	0.001
HBA	Hemoglobin subunit alpha	−0.31	0.001
QSOX1	Quiescin Q6	0.31	0.001
LRG1	Leucine-rich alpha2-glycoprotein 1	−0.30	0.002
C9	Complement component 9	−0.30	0.002
SERPINA10	Serpin peptidase inhibitor, clade A (alpha1 antiproteinase, antitrypsin), member 10	−0.30	0.002
ALP	Alkaline phosphatase, liver/bone/kidney	0.29	0.003
	mixed fc-gamma receptor III-A/B	0.29	0.003
PROC	Protein C (inactivator of coagulation factors Va and VIIIa)	−0.28	0.003
VCAM1	Vascular cell adhesion molecule 1	0.28	0.003
GAPDH	Glyceraldehyde-3-phosphate dehydrogenase	−0.28	0.004
OMD	Osteomodulin	0.27	0.006
IGKVD41	Immunoglobulin kappa variable 41	−0.27	0.006
IGFBP6	Insulinlike growth factor binding protein 6	0.26	0.007
PTPRG	Protein tyrosine phosphatase, receptor type, G	0.26	0.008
S100A9	S100 calcium binding protein A9 (calgranulin B)	−0.26	0.008
VNN1	Vanin 1	−0.26	0.008
SERPIND	Serpin peptidase inhibitor, clade D (heparin cofactor), member 1	−0.26	0.009
CA1	Carbonic anhydrase I	−0.25	0.009
CTSD	Cathepsin D (lysosomal aspartyl peptidase)	−0.25	0.01
HP	Haptoglobin	−0.25	0.011
SELENBP1	Selenium binding protein 1	−0.25	0.011
ORM2	Orosomucoid 2	−0.25	0.012
PRDX2	Peroxiredoxin 2	−0.25	0.012
AOC3	Amine oxidase, copper containing 3 (vascular adhesion protein 1)	0.25	0.012
COL6A3	Collagen, type VI, alpha 3	0.24	0.012
	Unidentified protein	−0.24	0.013
PZP	Pregnancyzone protein	−0.24	0.013
COL6A1	Collagen, type VI, alpha 1	0.24	0.014
PARK7	Parkinson disease (autosomal recessive, early onset) 7	−0.24	0.014
THBS1	Thrombospondin 1	−0.24	0.015
CAT	Catalase	−0.24	0.016
LCP1	Lymphocyte cytosolic protein 1 (Lplastin)	0.23	0.018
AFM	Afamin	−0.23	0.021
HPR	Haptoglobinrelated protein	−0.22	0.021
SELL1	Selectin L (lymphocyte adhesion molecule 1)	0.22	0.023
ENG	Endoglin	0.22	0.023
PFN1	Profilin 1	−0.22	0.026
PI16	Peptidase inhibitor 16	0.22	0.026
SERPINA6	Serpin peptidase inhibitor, clade A (alpha1 antiproteinase, antitrypsin), member 6	0.21	0.028
	Unidentified protein	−0.21	0.03
F9	Coagulation factor IX	−0.21	0.03
PROCR	Protein C receptor, endothelial	0.21	0.031
ORM1	Orosomucoid 1	−0.21	0.031
NEO1	Neogenin homolog 1	0.21	0.032
MMRN2	Multimerin 2	0.21	0.033
LGB	Beta-lactoglobulin	−0.21	0.034
CNTN4	Contactin 4	0.21	0.035
SHBG	Sex hormonebinding globulin	0.20	0.038
CA2	Carbonic anhydrase II	−0.20	0.043
IGFBP5	Insulinlike growth factor binding protein 5	−0.20	0.045
PLTP	Phospholipid transfer protein	0.20	0.046
FGA	Fibrinogen alpha chain	−0.20	0.046
	Unidentified protein	0.19	0.05
TPM4	Tropomyosin 4	−0.19	0.05
			
DiscoveryMAP Markers		Correlation to MHFMS	
Protein	Name	R-value	p-value
MB	Myoglobin	0.57	<0.001
SPP1	Osteopontin	0.54	<0.001
AXL	AXL receptor tyrosine kinase	0.44	<0.001
APSC	Amyloid P component, serum	−0.42	<0.001
CRP	C-reactive protein, pentraxinrelated	−0.41	<0.001
CCL22	Chemokine (C-C motif) ligand 22 (macrophage derived chemokine)	−0.41	<0.001
THBD	Thrombomodulin	0.40	<0.001
CALCA	Calcitonin	−0.40	<0.001
LEP	Leptin	−0.40	<0.001
NPPB	Brain natriuretic peptide b	0.37	<0.001
MMP2	Matrix Metalloproteinase 2	0.37	<0.001
CK	Creatine kinase muscle/bone	0.36	<0.001
ACE	Angiotensin converting enzyme	0.36	<0.001
FAPB3	Fatty acid binding protein (heart)	0.35	<0.001
CD40	CD40 Ligand	−0.34	<0.001
MIF	Macrophage Migration Inhibitory Factor	−0.34	<0.001
ANGPT2	Angiopoietin 2	−0.33	<0.001
AHSG	Alpha-2-HS-glycoprotein (fetuin A)	−0.33	0.001
CFH	Complement factor H	−0.33	0.001
IL8	Interleukin 8	−0.32	0.001
C3	Complement component 3	−0.32	0.001
PPY	Pancreatic polypeptide	0.31	0.001
VEGFA	Vascular endothelial growth factor	−0.30	0.002
TF	Transferrin	−0.29	0.002
PGF	Placental growth factor	0.29	0.002
EGF	Epidermal growth factor	−0.29	0.002
GSTA1	Glutathione S transferase alpha	−0.29	0.002
SOD1	Superoxide dismutase 1	−0.29	0.003
VCAM1	Vascular cell adhesion molecule 1	0.28	0.003
PAI1	Plasminogen activator inhibitor 1	−0.28	0.004
CSF1	Macrophage colony stimulating factor 1	0.28	0.004
S100A12	S100 Protein A12	−0.28	0.004
VTN	Vitronectin	−0.27	0.004
FASLG	Fas ligand	0.26	0.006
A1M	Alpha-1-microglobulin	−0.26	0.007
AST	Astartate transaminase	0.25	0.009
ACCT	Alpha-1-antichymotrypsin	−0.25	0.01
CCL3	Chemokine (C-C motif) ligand 3 (Macrophage Inflammatory Protein 1 beta)	−0.25	0.011
SORT1	Sortilin	−0.24	0.013
TBG	Thyroxine binding globulin	−0.24	0.014
APOA1	Apolipoprotein A1	0.24	0.015
MPO	Myeloperoxidase	−0.23	0.016
B2M	Beta 2 microglobulin	0.23	0.016
EPO	Erythropoietin	−0.23	0.017
MMP10	Matrix Metalloproteinase 10	−0.23	0.02
PROS1	Vitamin K Dependent Protein S	−0.22	0.023
MMP7	Matrix Metalloproteinase 7	−0.22	0.025
AGER	Advanced glycosylation end products receptor	0.21	0.029
IL18	Interleukin 18	0.21	0.033
CCL11	Chemokine C-C motif ligand 11	−0.21	0.034
IGA	Immunoglobulin A	−0.20	0.035
C peptide	Proinsulin C Peptide	−0.20	0.041
A2M	Alpha-2-macroglobulin	−0.20	0.041
PDGF BB	Platelet Derived Growth Factor	−0.20	0.042
CCL16	Chemokine C-C motif ligand 16	−0.19	0.047
IL1A	Interleukin 1 alpha	0.19	0.049
APOA4	Apolipoprotein A4	0.19	0.049
MMP9	Matrix metalloproteinase 9	−0.19	0.05
			
OncologyMAP Markers		Correlation to MHFMS	
Protein	Name	R-value	p-value
SPP1	Osteopontin	0.53	<0.001
CLEC3B	Ctype lectin domain family 3, member B (tetranectin)	0.51	<0.001
IGFBP6	Insulin-like growth factor binding protein 6	0.48	<0.001
FABP4	Fatty acid binding protein (adipocyte)	−0.45	<0.001
CHI3L1	Chitinase 3-like 1 (YKL-40)	−0.41	<0.001
LEP	Leptin	−0.39	<0.001
CTSD	Cathepsin D	−0.33	0.001
MST1	Macrophage stimulating 1 (hepatocyte growth factor-like)	−0.33	0.001
MIF	Macrophage migration inhibitory factor	−0.32	0.001
S100A4	S100 calcium binding protein A4	−0.32	0.001
GLO1	Glyoxalase 1 (lactoylglutathione lyase)	−0.32	0.001
ENG	Endoglin	0.30	0.001
FTL1	Fms-related tyrosine kinase 1 (vascular endothelial growth factor receptor)	−0.30	0.002
ERBB2	Human epidermal growth factor receptor 2 (HER2)	−0.28	0.003
NDKB	Nucleoside phosphatase kinase isoform B	−0.28	0.004
PRDX-4	Peroxiredoxin 4	−0.25	0.01
PLAUR	Plasminogen activator, urokinase receptor	−0.24	0.015
IL6R	Interleukin 6 receptor	0.23	0.02
CCL24	Chemokine (C-C motif) ligand 24 (eotaxin 2)	−0.21	0.034
GSN	Gelsolin	0.20	0.038
PSAT1	Phosphoserine aminotransferase 1	−0.20	0.039
TGFB1	Transforming growth factor beta 1	−0.19	0.049

Markers that regressed to MHFMS SMA motor scores from the BforSMA study are listed by each analysis with their R-values and p–values. TNXB appears twice due to the positive regression with two unique isoforms.

Next, we used the BforSMA samples to probe for new motor function markers in ready-made Luminex panels in multiplex format (DiscoveryMAP v1.0® and OncologyMAP v1.0® by Myriad RBM, a total of 233 analytes). Analysis of BforSMA samples in DiscoveryMAP v1.0® and OncologyMAP v1.0® identified 51 and 13 new motor function associated biomarker candidates respectively (Table 1). Plasma concentrations for each protein of interest were analyzed for regression to the MHFMS for each subject. 14 analytes present in the MAPs were identified as markers with statistically significant association with motor function in the LC/MS study –11 of these also significantly associated with motor function in the MAP analysis (Table 2). Of the analytes that could not be reproduced as motor regressors, IGFBP5 and SHBG gave marginally significant LC/MS p-values of 0.045 and 0.038. HP also did not repeat, possibly due to sample processing differences between the LC/MS and immunoassay platforms.

**Table 2:** BforSMA LC/MS and MAP Repeat Hit Rankings.

	LC/MS	DiscoveryMAP v1.0®	OncologyMAP v1.0®
APCS	14	4	N/A
APOA4	22	57	N/A
CTSD	49	N/A	7
CRP	17	5	N/A
ENG	66	N/A	12
GSN	19	N/A	20
HP	50	N/A	89 (non-hit)
IGFBP6	43	N/A	3
SPP1	*	2	N/A
THBS1	60	103 (non-hit)	N/A
VCAM1	39	29	N/A
VTN	15,23	33	N/A
SHBG	78	75 (non-hit)	N/A
IGFBP5	80	N/A	50 (non-hit)

Analytes that were motor function regressor hits in the LC/MS campaign that were represented on the MAP panels were ranked side-by-side. The majority of markers were repeat hits, while some did not repeat (non-hit).

* indicates that marker SPP1 was a strong hit analyte in the original analysis of BforSMA hits using different statistical methods. VTN appears twice in the LC/MS analysis due to the presence of multiple hit isoforms.

We examined the association of the biomarkers with MHFMS scores, SMN2 copy number, SMN protein levels, and quantity of SMN2 full length, SMN full length, SMN7 delta, and total SMN transcripts. The analyses included univariate methods and multivariate regression methods (linear regression, lasso, stepwise regression, and random forest).

We selected 35 biomarkers associated with one or more dependent variables based on statistical significance, importance in random forest models, and non-statistical criteria such as assay performance, distribution of values near or below the lower limits of quantitation or detection and known biological relationships (Table S2). Other criteria for selection included performance of assays and long-term availability of reagents. This pilot 35 biomarker set included the 8 biomarkers chosen from the LC/MS campaign for new assay development. The 35 biomarkers chosen for validation were present on pre-existing multiplexes that included an additional 91 proteins, so these additional analytes were also examined.

### Validation Phase: PNCR Natural History Study

The 35 putative SMA biomarkers from the discovery phase were evaluated in an independent cohort of SMA patients: a natural history study (NHS) by the Pediatric Neuromuscular Clinical Research (PNCR) network that included subjects with more severe disease and from a broader range of ages than BforSMA (0.25–45 years, versus 2–12 years) [15], [22].

**Figure 2:**
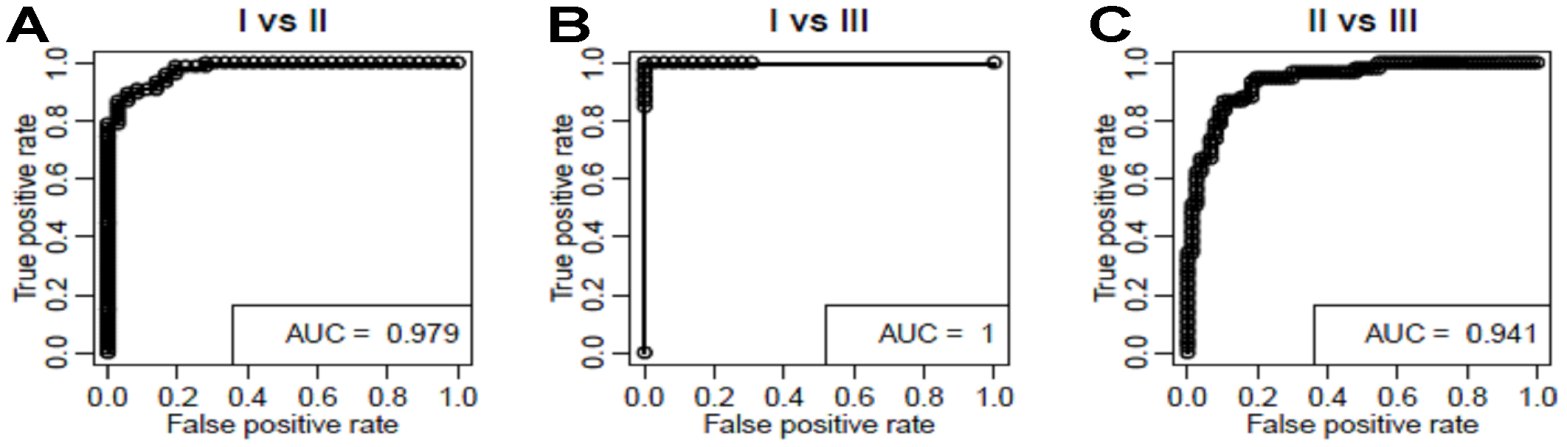
Classification of SMA types by the top 13 biomarker analytes Receiver-Operator curves. (ROCs) and (area under the curves (AUCs) were generated for the top 13 markers to differentiate between SMA types within the PNCRN’s natural history study dataset. Both sensitivity (True positive rate) and specificity (1-False positive rate) of the SMA type classifications were very high across several thresholds. **A:** Type 1 versus Type 2 AUC was 0.98. **B:** Type 1 versus Type 3 AUC was 1. **C:** Type 3 versus Type 3 AUC was 0.94.

We tested the 35 analytes for relationships with motor function measures (HFMS). In linear regression analyses of the 35 candidates, we found 12 significantly associated (p<0.05) with motor function and a 13^th^ with a p-value of 0.058 (Table 3). The set of 13 top analytes includes APCS, AXL, CD93, CDH13, CHI3L1, COMP, DPP4, LEP, LUM, MB, PEPD, SPP1, and THBS4. These 13 analytes became candidates for inclusion in the Tobit regression model to predict HFMS, described below [27]. These 13 showed similar regression results in analyses of other motor function endpoints (HFMSE, GMFM, CHOP-TOSS and highest motor function). Neither weight, height, age at clinic visit, age at enrollment, nor SMN2 transcript or protein levels, was found to be important clinical covariates to the regression results. However age of onset was found to be an important clinical covariate and was included in the Tobit models to predict motor scores described below. The 13 biomarkers were also combined in a logistic regression model to discriminate among SMA types using a receiver-operator curve (ROC) analysis; AUCs for classification of SMA type ranged from 0.94 to 1 (Figure 2).

**Table 3:** Top 13 SMA motor function regressors are markers in two SMA populations.

	Correlation to MHFMS			
	PNCR NHS values		BforSMA values	
Protein	R-value	p-value	R-value	p-value
COMP	0.526	<0.001	0.519	<0.001
AXL	0.5	<0.001	0.414	<0.001
CD93	0.487	<0.001	0.416	<0.001
PEPD	0.485	<0.001	0.444	<0.001
THBS4	0.444	<0.001	0.523	<0.001
LUM	0.423	<0.001	0.427	<0.001
MB	0.401	0.001	0.573	<0.001
DPP4	0.397	0.001	0.375	<0.001
SPP1	0.382	0.002	0.536	<0.001
CHI3L1	−0.314	0.01	−0.426	<0.001
CDH13	0.255	0.039	0.329	0.001
APCS	−0.252	0.041	−0.422	<0.001
LEP	−0.235	0.058	−0.384	<0.001

The top 13 list was compiled based on significant regression to HFMS and other SMA motor outcome measures in the PNCRN natural history study sampleset. The pilot panel of markers tested included 35 top motor analytes identified via the BforSMA study and these 13 analytes represent robust repeat markers in a distinct SMA population.

The association of the 35 biomarkers with non-motor SMA outcome measures was also examined: pulmonary function (FVC), electrophysiology (CMAP and MUNE), quality of life (PedQL), and myometric strength measures of elbow flexion (MyoEF), elbow extension (MyoKE), and knee flexion (MyoKF). The top 13 motor function biomarkers were in general poorly associated with the non-motor outcomes with R^2^-values ranging from 0 to 0.36. However, other analytes were associated with non-motor outcomes; these were either previously identified as motor function markers in BforSMA, or were altogether novel markers (Table 4). In general, analyte relationships to the non-motor outcomes were less strong and also less numerous than those for the motor function regressors (Table 1, Table S4). One notable exception to this observation was pulmonary function, as adjusted R^2^ for FVC was as high as 0.62. Overall this may lend confidence to our approach in using motor function, as motoric changes specifically are what typify SMA disease progression.

**Table 4:** SMA non-motor outcome regressors.

Outcome Measure	Analyte	R-value	p-value
CHOP-TOSS	ERBB2	0.597	0.002
	IL10	0.432	0.035
	IL18	0.422	0.04
	IGFBP1	0.41	0.046
	GOT1	0.412	0.046
	INS	−0.405	0.049
FVC	MB	0.52	0
	TNFR2	0.432	0.001
	A2M	−0.374	0.005
	CCL4	0.339	0.011
	RSTN	0.328	0.013
	TNFRI	0.33	0.013
	IGFBP5	−0.323	0.015
	FRTN	0.306	0.022
	UPA	−0.303	0.023
	pINS	0.3	0.024
	AXL	0.273	0.042
	TIMP1	0.27	0.044
	FBLN1C	−0.264	0.049
	IL10	0.264	0.049
CMAP	DPP4	0.261	0.022
	ADIPOQ	−0.256	0.025
	APOB	−0.252	0.027
	pINS	0.246	0.031
	CCL4	−0.245	0.032
	LUM	0.244	0.033
	PPY	0.238	0.037
	IL12P40	−0.236	0.039
	MMP7	−0.236	0.039
	VWF	0.236	0.039
	PEPD	0.226	0.048
MYOEF	MB	0.455	<0.001
	PEPD	0.385	0.002
	pINS	0.298	0.018
	A2M	−0.274	0.031
	DPP4	0.274	0.031
	pINS (total)	0.271	0.033
MYOKF	pINS	0.398	0.002
	pINS (Total)	0.345	0.008
	MB	0.311	0.017
	INS	0.306	0.019
	IGFBP1	−0.27	0.038
	PEPD	0.27	0.038
	DPP4	0.261	0.046
	COMP	0.259	0.047
MYOKE	COMP	0.421	0.001
	LUM	0.41	0.001
	TG	−0.394	0.002
	PEPD	0.38	0.003
	MB	0.36	0.005
	DPP4	0.321	0.012
	FBLN1C	−0.324	0.012
	APOB	−0.32	0.013
	PGI	−0.32	0.013
	MMP9	−0.297	0.021
	ANG	−0.292	0.024
	THBS4	0.278	0.032
	TNXB	−0.276	0.033
	MSP	−0.269	0.038
	CD93	0.264	0.042
	FRTN	0.263	0.043
PQP	AXL	0.394	0.001
	DPP4	0.297	0.012
	ADIPOQ	−0.294	0.013
PQC	CD93	0.456	0.001
	FASLR	0.424	0.002
	CLEC3B	0.367	0.007
	INS	0.27	0.05

Several markers were identified in the PNCR NHS as regressing to the Children’s Hospital of Philadelphia Test of Strength (CHOP-TOSS), pulmonary function (best FVC), electrophysiology (CMAP and MUNE), strength as measured by knee and elbow flexion and extension(log average MyoEF, MyoKE, MyoKF), parent-reported and child-reported quality of life (PQP and PQC).

### Biomarker Panel: SMA-MAP

27 analytes were selected for inclusion into a new biomarker panel, called SMA-MAP (Table 5). The 13 analytes that regressed to motor outcomes of SMA in both the BforSMA and PNCR NHS studies were included. An additional 12 SMA-MAP analytes (AHSG, APOB, CCL2, CFH, CLEC3B, CRP, CTSD, ENG, ERBB2, FBLN, IFBP6, PGF, TNXB) were motor regressors from the BforSMA analysis and/or related to non-motor outcomes. Lastly, IGF1 was included due to the reported disruption of the IGF pathway in SMA models and human muscle as well as interest in IGF1 therapy for SMA [28]–[32]. The SMA-MAP panels were assembled to minimize sample volume requirements, requiring only 100 µL per sample for analysis, and met multiplex assay validation acceptance criteria. SMA-MAP analytes were verified in a multiplex, and tested for the fundamental assay parameters of lowest detectable dose, precision, cross-reactivity, linearity, spike-recovery, dynamic range, matrix interferences, freeze-thaw stability and bench-top stability (Table S3).

**Table 5:** SMA-MAP analytes and their correlated outcomes.

Analyte	Correlated Outcome Measures
APOB	CMAP, Strength
APCS	Motor
ASHG	Motor, PD, CMAP, Strength
AXL	FVC, Motor, PD?, PQP
CCL2	PD?
CD93	Motor, MUNE, PQC, Strength
CFH	Motor
CDH13	Motor
CHI3L1	Motor
CLEC3B	Motor, PQC
COMP	Motor, MUNE, Strength
CRP	Motor, PD?
CTSD	Motor
DPP4	Motor, MUNE, PQP, Strength
ENG	Motor, PD?
ERBB2	CHOP-TOSS, Motor, PD?
FBLN1	FVC, Motor, PD?, Strength
IGF1	PD?
IGFBP6	Motor, PD?
LEP	Motor
LUM	CMAP, Motor, MUNE, PD? Strength
MB	FVC, Motor, Strength
PEPD	CMAP, Motor, MUNE, Strength
PGF	Motor, MUNE, PD?
SPP1	Motor
THBS4	Motor, MUNE, PD?
TNXB	Motor

CHOP-TOSS = Children’s Hospital of Philadelphia Test of Strength, FVC = forced vital capacity, Motor = motor function scale (e.g. MHFMS, HFMS, HFMSE, GMFM), PD = SMN pharmacodynamic measure, CMAP = compound motor action potential, MUNE = motor unit number estimation, PQP = PedQL quality of life, parent score, PQC = PedQL quality of life, child score, CHOP-TOSS = Children’s Hospital of Philadelphia Test of Strength.

Unthawed BforSMA aliquots were re-analyzed using SMA-MAP to compare its analyte values to the initial values generated from DiscoveryMAP v1.0®, OncologyMAP v1.0®, and the new assays created for the 8 LC/MC hit analytes.

### Regression Model to Predict Motor Scores

**Figure 3:**
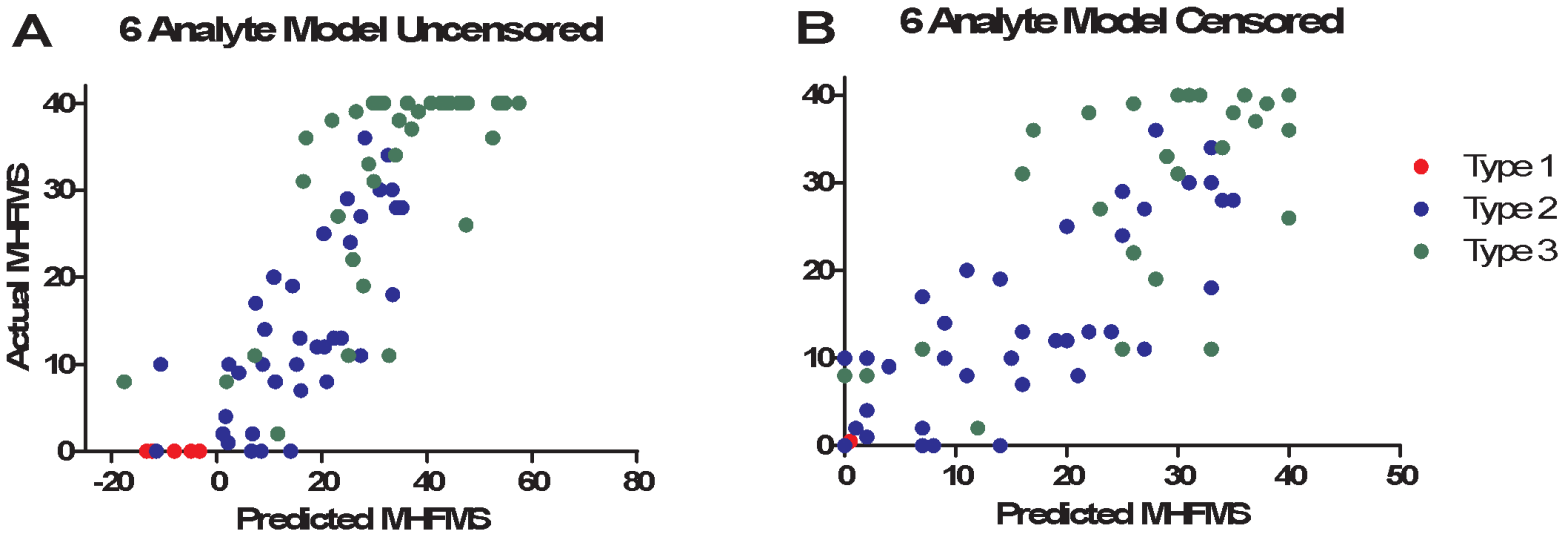
SMA-MAP motor function score prediction model. Using Tobit linear regression models SMA motor scores were predicted from SMA-MAP analytes values with age of onset as a covariable. Pearson correlations between actual and predicted motor scores for the top 6 combinations from BforSMA were plotted. **A**: Graph of actual and predicted motor scores of a 6 analyte model uncensored model. Type 1 SMA patients and ambulatory Type 3 subjects can be represented in the analysis and given a score below 0 or over 40 respectively. **B**: Graph of 6 analyte motor scores using values censored between 0 and 40. Note that the Type 1 datapoints have been moved arbitrarily to the right to allow visualization, and these points still represent values of 0.

We developed a Tobit regression model to predict MHFMS scores using SMA-MAP biomarker values, based on the MHFMS framework and age of onset information from BforSMA. As noted above, age of onset was found to be an important clinical covariate in the linear regression analyses for both BforSMA and the PNCR NHS studies, and thus was included in the Tobit modeling. We selected the final model by testing all possible subsets of the top 13 analytes, with data from SMA type 1, 2, and 3 subjects from the BforSMA study. All 13 analytes were entered as candidates in the models. Performance of the models was compared using adjusted Pearson R^2^ values between actual and predicted motor scores calculated on bootstrap (out-of-bag) samples. Six analytes (APCS, COMP, DPP4, LEP, MB, and THBS4) produced a model with the highest bootstrap out-of-bag correlations of predicted with actual motor scores. Many alternative models with different subsets of the analytes gave similar our-of-bag performance. The correlation between actual and predicted BforSMA scores with the 6 analytes was R = 0.89 for scores censored between 0–40 and R = 0.86 for uncensored scores (Figure 3). Separate models were created to predict scores within the 0–40 numeric range, and one with uncensored scores. Coefficients for the motor score regression model for the 6 SMA-MAP analytes and age of onset as well as an Excel-based version of the predictive tool are available for download (http://neuinfo.org/smabiomarkers/).

## Discussion

The use of plasma protein biomarkers for cardiovascular disease and cancer has been transformative in advancing new drug development and improving care management. SMA could also potentially benefit from new biomarkers, as several new drugs are in or poised to enter clinical trials [1]. SMA is a rare pediatric disease with significant unmet medical need, a heterogeneous presentation and a disease course punctuated by irreversible events (e.g. loss of the ability to walk). Thus it is vital to validate biomarkers that could help shorten the length of drug trials, stratify patient populations, and allow for smaller study sizes. Molecular biomarkers are valuable complements to clinical SMA motor outcome measures that are subject to age limitations and motivation for performance [10]. Also, while several SMN-based pharmacodynamic (PD) biomarkers for SMA exist, not all trials will test SMN-upregulating drugs, and other non-SMN markers would be needed [21], [25], [33]. In addition, not all therapeutic interventions will be delivered systemically, and use of a biomarker matrix like plasma that reflects proteins from a number of sites may provide additional insights over measures based in blood cells [33].

**Figure 4:**
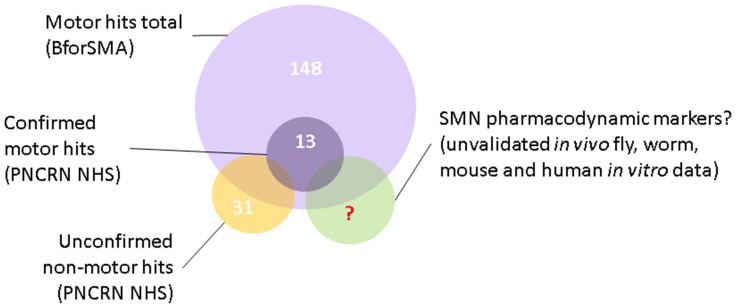
Several types of biomarkers for SMA-MAP analytes. SMA biomarkers were identified for their regression to motor function and non-motor outcome measures. The analytes have been confirmed and validated to different degrees and will require more validation in prospective, longitudinal studies to determine their utility as biomarkers for disease progression and pharmacodynamic response.

Here we describe the development of a biomarker panel (SMA-MAP) for plasma proteins in SMA patients associated primarily with motor function and confirmed in multiple patient populations from infancy to adulthood that can be used to help evaluate the current neuromuscular status of patients. The work described here advances the prior publication on candidate plasma protein biomarkers discovered by mass-spectrometry proteomics in the BforSMA study in a number of ways: by reanalyzing the LC/MS results in the context of SMA patients only, validating a subset of those analytes and identifying new putative markers in a different platform, as well as confirming the strongest markers in a new SMA cohort. The resulting SMA-MAP panel can accurately classify SMA patients by type, generate predicted motor function scores, and a subset may have relationships to pathways linked to SMN or associate with non-motor outcome measures (Figure 4).

In the discovery phase with BforSMA samples analytes were probed for relationships to SMA motor function. We employed off-the-shelf biomarker panels from Myriad RBM that required modest volumes of plasma to advance our analyses quickly, and built new assays for the strongest LC/MS discovery hits not represented in panels. In the validation phase we tested PNCR samples to confirm our results, choosing to delay selecting a small number of analytes until further analysis. A large pilot panel of 126 analytes confirmed prior results with our top 35 motor markers. Also we identified candidate markers for non-motor outcomes that figure to be important secondary outcome measures for SMA trials – all of which require confirmation in another patient collection (Figure 1). While some of these candidate markers identified also regressed to motor function, several others associated with non-motor outcome measures like electrophysiology, muscle strength, pulmonary function, and quality of life were novel. Data from all phases of analysis were used to assemble a panel of 27 analytes, filtering by strength of regression to motor function and other outcome measures, and assay performance.

SMA-MAP complements established clinical outcome measures and markers, and has novel advantages and benefits. The tool can generate predicted motor scores based on the framework of the MHFMS. Correlation values of predicted and actual motor scores were relatively high, with adjusted R^2^-values values reaching 0.56 in multivariate modeling when age of onset was used as a covariate (Table S4). The importance of age on the SMA biomarker regression to clinical outcome measures echoes relationships between age and SMN transcript in the BforSMA study and disease duration and motor function reported by Tiziano et al. [25], [34]. While motor regression values were high for the SMA-MAP motor analytes, imperfections in the correlations themselves may be valuable, as actual motor scores from SMA children are subject to motivation, while values from a biochemical panel are more objective. Tobit model motor prediction can also range below and above motor scale floor and ceiling values, allowing prediction of motor function in type 1, 2 and 3 SMA patients using the same tool [27]. Also, the SMA-MAP allows evaluation in subjects who are younger than are usually testable in motor scales (30 months) [10].

While the regression values of the panel analytes to motor function are strong and top analytes were confirmed in different cohorts, there are limiting aspects to our approach. One obvious weakness is that SMA-MAP is based on plasma analytes whereas SMA is a disease in which muscle and spinal motor neurons are the most affected tissues and cells. Tissue samples (e.g. muscle biopsies) were not collected in the BforSMA and thus no relationships between neuromuscular disease-relevant tissues and the SMA-MAP were assessed. It should be noted however, that SMA patients and models have other non-neuromuscular disease features as well, including potential metabolic syndromes [35], [36]. Use of plasma could potentially be advantageous, as achieving consistent and high-quality sampling with plasma is more straightforward than with muscle – which has been shown to generate markedly different biomarker signatures and denervation patterns in SMA mice depending on sampling site [37], [38].

Another notable deficit in the generation of SMA-MAP is that it did not rely heavily on longitudinal sampling in its development. The BforSMA study that generated samples for the majority of the discovery process was a single-visit clinical study, while only baseline and 12 month visits were assessed in the PNCR study. There were only N = 55 PNCR study subjects represented at both timepoints in the validation experiment – a number too small to generate a meaningful statistical analysis. As a result, though these biomarkers may classify SMA severity across a spectrum of motor phenotypes, they are not classical biomarkers of progression. As with any potential biomarker, these limitations will not be remedied and their utility will not be expanded upon without significant additional work by the greater research community.

Many SMA-MAP markers are pleiotropic connective tissue, extracellular matrix, and growth factor pathway proteins that play roles in neural development, injury, and maintenance [39], [40]. While it is tantalizing to imagine these markers are signals to similar processes in SMA, their relationship to underlying disease biology is unclear. The top 13 markers are also biomarkers of Ehlers-Danloss (TNX), and juvenile, osteo- and rheumatoid arthritis, all of which have feature connective tissue damage and abnormal mobility of joints. (CLEC3B, COMP, LUM, SPP1) [41]–[44]. Aside from lowered bone fracture thresholds due to an inability to bear weight, there is also some evidence that SMN interacts with bone proteins [45], [46]. Other markers like CDH13, DPP4, IGF1, and LEP are involved in control of body composition, growth, and insulin regulation, all of which are either altered in SMA or being more actively explored [35], [47]–[52]. Molecularly, this may be of some interest, as both severe growth failure in some forms of primordial dwarfism and SMN deficiency are associated with reductions in components and activity of the minor spliceosome (Lotti 2012, He 2011). Lastly, many of these markers have been identified in oncology studies. There are no data on cancer in SMA, but there are reports that SMN interacts with proteins involved with promoting entry into the cell cycle and stem cell proliferation, and that SMN is highest in mammalian tissues with greater regenerative capacity [53]–[55].

Some top analytes (e.g. AXL, CLEC3B, COMP, ERBB2, IGF1, PGF, SPP1) could be SMA PD markers of drug-induced SMN changes. These markers and many others in the biomarker candidate list (Table 1) are members of RTK/mTOR, STAT, TGFB/SMAD/BMP or p21 pathways that have been published in SMA cellular, invertebrate, and mouse models as modulators of disease phenotype and SMN [28], [29], [56]–[60]. Some analytes probed had been identified as possible SMA therapeutic targets or biomarkers: CCL2, GLO1, IGF1, and PRL [28]–[31], [61], [62]. These potential PD markers require analysis in an SMN-upregulating *in vivo* treatment paradigm.

Our unbiased biomarker identification plan raises uncertainties about which markers are specific to SMA or are common to secondary neuromuscular degeneration. Indeed, some SMA-MAP analytes are themselves markers or members of biological networks implicated for Amyotrophic Lateral Sclerosis (ALS), Duchene Muscular Dystrophy (DMD) or other neurodegenerative diseases: CCL2 and ENG for ALS, LUM and SPP1 for DMD, and CRP, CTSD, and IGF1 for Parkinson’s and Alzheimer’s [60], [63]–[75]. While biomarkers specific to SMA could also help shed light on disease biology and perhaps identify new therapeutic targets, our goal was to identify and confirm biomarkers sensitive to patient status regardless of specificity to SMA. We do recommend more comprehensive pathway analyses be performed on these biomarkers. These studies could include analysis of disease-relevant SMA tissue, mouse model studies with drug treatment, and also analysis of other disease control analysis with plasma from other neuromuscular and neuropathic disorders. These could include ALS, DMD, and congenital myotonic dystrophies, particularly ones like Nemaline and Central Core CMD that are similar to SMA in that they lack necrotic and fibrotic muscle atrophy features. Indeed if the panel is not specific to SMA and shows utility in other diseases, it will remain useful for research for assessing disease stage in confirmed SMA cases.

There are also caveats and potential areas for further investigation related to the statistical methods. In any situation in which the number of analytes is relatively high compared to the number of samples being analyzed, there is a risk of overestimating the strength of statistical relationships. To mitigate this, we modeled the association with 100 rounds of multivariate bootstrapping and represented the output in adjusted R-values, which penalizes the number of variables in the models. The motor prediction tool operates within the framework of the MHFMS or the HFMS, but could be modified for other motor scales like the GMFM or HFMSE. The motor prediction tool itself could be expanded with additional modeling and also data from future analyses. Work is ongoing to build an accessory tool to classify SMA into types akin to Srivivasta et al. by using machine learning and other models [76]. Such a tool could be used in trials or clinical practice to track a patient’s ‘type’ over time to assess whether they are transitioning towards a more severe or mild phenotype.

In summary, the SMA-MAP is the culmination of a biomarker discovery campaign testing nearly 1000 plasma proteins, performed in multiple patient sample sets and quantitation technologies, and is ready for further validation to determine the extent of its utility in clinical research and trials. Ongoing and future work includes testing the panel with samples and data from interventional studies as well as in new longitudinal SMA natural history studies, such as the one proposed for SMA in the NeuroNEXT initiative [77]. Determining whether some SMA-MAP markers are both motor function and PD markers remains an important next step. This exploration will proceed in SMA animal models, and also hopefully in new drug trials. The tool and its motor prediction algorithm offer quantitative and objective evaluations that may become valuable additions to the SMA clinical research community.

## Materials and Methods

### Ethics Statement

Healthy control and SMA patient samples from the BforSMA study were collected in accordance with protocols approved by a central Institutional Review Board (IRB) (New England Research Institutes, Inc. Institutional Review Board) as well as by each sites’ IRB before enrollment at that site (Children’s Hospital of Boston Institutional Review Board, Office of Clinical Investigation; The Children’s Hospital of Philadelphia Institutional Review Board; Cincinnati Children’s Hospital Medical Center Institutional Review Board; Colorado Multiple Institutional Review Board - University of Colorado Denver Institutional Review Board; Columbia University Medical Center Institutional Review Board; Johns Hopkins Medical Institutional Review Board; Mayo Clinic Institutional Review Board; New England Research Institutes, Inc. Institutional Review Board; The Ohio State University Biomedical Institutional Review Board; Research Ethics Board for The Hospital for Sick Children; Stanford University Institutional Review Board; University of Alabama at Birmingham Institutional Review Board for Human Use; University of Iowa Institutional Review Board; The University of Utah Institutional Review Board; The University of Western Ontario Research Ethics Board for Health Sciences Research; University of Wisconsin Health Sciences Institutional Review Board; The UT Southwestern Institutional Review Board; Washington University in St. Louis Institutional Review Board; Wayne State University Institutional Review Board, Human Investigation Committee). Written informed consent for participation was obtained from the legal guardians of all subjects and assent for participation was obtained directly from subjects whenever applicable. S for children over 7 years of age [26].

Data and samples from SMA patients in the natural history study by the Pediatric Neuromuscular Clinical Research (PNCR) Network were collected under the auspices of the protocols approved by each site’s IRB: Columbia University, The Children’s Hospital of Philadelphia and the University of Pennsylvania, and Harvard University [15], [22]. Written informed consent or verbal assent was provided and recorded by PNCR staff on IRB-approved documents for all PNCR parents or participants in the natural history study, which allows for subsequent analysis with study data and materials upon approval by the PNCR Biorepository Committee. Materials and data from the PNCR NHS were made available following approved of a written request to the PNCR Biorepository managed by Dr. Wendy Chung at Columbia University. All BforSMA and PNCR NHS data were de-identified and analyzed anonymously.

### Platforms, Study Plasma Samples and Data

Data were generated across multiple discovery campaigns and platforms using samples from different SMA studies. The first platform was a LC/MS iTRAQ with analysis performed by BG Medicine. The second platform was comprised of multiplexed immunoassays using the Luminex system. Analysis with the DiscoveryMAP v1.0® and OncologyMAP v1.0® panels, as well as a 126 analyte pilot panel (containing the top 35 motor regressor analytes from the tested MAPs) and the 27 analyte SMA-MAP were all performed by Myriad RBM on the Luminex platform. 8 new Luminex immunoassays were created for top analytes that regressed to motor function in the LC/MS campaign, and are included in both the 126 analyte pilot panel and the SMA-MAP. CILP2 and ADAMTSL4 were initially chosen for new assays but were discarded due to poor reagent availability and assay performance. Samples from the PNCR natural history study were analyzed in the 126 analyte pilot panel.

The BforSMA study was a multi-center, pilot study enrolling 130 subjects, aged 2 to 12 years from 18 academic pediatric neuromuscular clinics [25], [26]. Each subject was seen for a single visit, during which an assessment of functional ability (Modified Hammersmith Functional Motor Scale, MHFMS), pulmonary status (forced vital capacity, FVC), and nutritional status was performed. There was no therapeutic intervention. Three groups of SMA patients and one cohort of control children were enrolled according to the following classifications in the BforSMA study: type I SMA (n = 17), type II SMA (n = 49), type III SMA (n = 42), healthy control children (n = 22). 129 plasma samples were collected from the SMA patients and matched control subjects.

The PNCR SMA natural history study (NHS) was conducted at Columbia University, Boston Children’s Hospital and Children’s Hospital of Philadelphia, with the Muscle Study Group at the University of Rochester serving as the data coordinating center [15], [22]. This NHS study was a multisite, longitudinal prospective study enrolling 101 patients aged 3 months to 45 years from three academic pediatric neuromuscular clinics. Subjects were assessed using multiple motor scales and tests (HFMS, Expanded-HFMS; Gross Motor Function Measure, GMFM; Children’s Hospital of Philadelphia Test of Strength, CHOP-TOSS). Secondary outcome measures included pulmonary status (forced vital capacity, FVC), strength (myometry for elbow and knee flexion, MyoEF and MyoKF; Myometry for knee extension, MyoKE), nerve/muscle physiology (compound motor action potential, CMAP and Motor unit number estimation, MUNE) and quality of life (PedsQL™ Parent and child scores). SMN1 and SMN2 were genotyped. Age of onset and highest motor function were also collected by parental or self-report. There was no therapeutic intervention. The 158 plasma samples for the pilot biomarker panel analysis included three SMA groups from the 0 and 12 month NHS visits: subjects with type 1 SMA (n = 27), type 2 SMA (n = 40) or type 3 SMA (n = 34). N = 55 subjects were represented at both timepoints (N = 9 type 1, N = 23 Type 2, and N = 23 type 3). PNCR patients in the biomarker analysis ranged in age from 0.25 to 45.1 years.

### Liquid Chromatography

We performed a statistical reanalysis of the data previously published by Finkel et al. in the BforSMA proteomics study; the authors previously generated their results using a mass-spec/mass-spec (MS/MS) combined with iTRAQ labeling [26]. Briefly, the plasma samples from the BforSMA study were depleted of high abundance proteins sequentially by using an IgY14 column and a supermix column (both by Sigma-Aldrich, St. Louis, MO). Samples were reduced (TCEP), alkylated (iodoacetate) and digested (trypsin) prior to 8-plex iTRAQ labeling. 6 of the 8-plex channels were used for primary individually tagged samples while the remaining 2 were a reference mixture pool of all BforSMA samples. The labeled samples were pooled and separated to 6 fractions using a strong cation exchange column. Fractions were further processed by high pressure liquid chromatography (HPLC), matrix-assisted laser desorption/ionization (MALDI), and MS/MS. The quantity of each protein analyte was represented by an average ratio of reporter ion intensities between the 6 primary sample channels and the 2 reference channels. Signal integration, analysis and normalization was done as described [26].

### Immunoassay Multi-Analyte Profile (MAP)

Multiplexing was accomplished by assigning each analyte-specific assay a microsphere set labeled with a unique fluorescence signature. Each set of microspheres are encoded with a fluorescent signature by impregnating the microspheres with a unique dye combination. After encoding, an assay-specific capture reagent is conjugated covalently to each unique set of microspheres, creating an ELISA-like assay on each bead surface. After optimizing the parameters of each assay separately, Multi-Analyte Profiles (MAPs) are performed by mixing up to 100 different sets of the microspheres in a single well of a 96- or 384-format microtiter plate. A small sample volume of plasma (10 uL–20 uL) is added to the well and allowed to react with the microspheres. The assay-specific capture reagent on each individual microsphere binds the analyte of interest. A cocktail of assay specific, biotinylated detecting reagents (e.g., antibodies), is reacted with the microsphere mixture, followed by a streptavidin-labeled fluorescent “reporter” molecule. Finally, the multiplex is washed to remove unbound detecting reagents. After washing, the mixture of microspheres is analyzed using the Luminex 100™ instrument. Each individual microsphere passing through the instrument’s excitation beams is analyzed for its encoded unique fluorescence signature and the amount of fluorescence generated in proportion to the analyte. As the microsphere passes through a green diode-pumped solid state laser (532 nm) and is identified by its signature, a fluorescence “reporter” signal (580 nm) is generated in proportion to bound analyte concentration.

### SMA-MAP Validation Testing

The least detectable dose (LDD) was determined by adding three standard deviations to the average of the signal for 20 replicate determinations of the standard curve blank. This value was converted to concentration as interpolated from the standard curve (LDD) and multiplied by the dilution factor used for testing plasma samples. The lower limit of quantification (LLOQ) was defined as the point at which the Coefficient of Variation (CV) for samples was 30%. It was determined by 2 fold dilutions of Standard 5 for 8 dilutions and assaying the samples in triplicate over three different runs. The CV was calculated and plotted against concentration. The LLOQ was interpolated from this plot, multiplied by the dilution factor. The dynamic range is the range of standard used to produce the dose response curve multiplied by the dilution factor. Precision (Intra- and Inter-Run) was determined by measuring 3 levels of controls (C1–C3) in triplicate over 5 runs and provides information concerning random error expected in a test result caused by person, instrument, and day variations. The acceptance criteria for precision is C1<25% and C2, and C3<20%. Acceptance criterion for most other metrics is an average value between 70–130% (linearity, spike recovery, interference, freeze-thaw, etc).

Cross-reactivity was determined by testing high concentrations of each single standard in the multiplex assay. Linearity is the ability of the assay to obtain test results that are proportional to the concentration of analyte in the sample when serially diluted to produce values within the dynamic range of the assay. Linearity was determined by normal human plasma and control level 3 serially diluted in sample dilution buffer throughout the assay range. The % recovery was calculated as observed vs. expected concentration.

Spike recovery is used to account for interference caused by compounds introduced from the physical composition of the sample or sample matrix that may affect the accurate measurement of the analyte. Spike recovery was performed by spiking different amounts of standard into the standard curve diluent (control spike) and known serum and plasma samples. The average % recovery was calculated as the proportion of spiked standard in the sample (observed) to that of the control spike (expected). The acceptance criteria for spike recovery are between 70–130% for a minimum of 3 out of 6 samples. The purpose of matrix interference is to determine whether the presence of substances commonly found in samples that may interfere with immunoassays introduce any systematic error in the multiplex. Matrix interference was determined by spiking Hemoglobin, Bilirubin, and Triglyceride into samples and determining % recovery as observed (spiked sample) vs. expected (unspiked sample). The purpose is to determine the ability of an antigen to tolerate freeze-thaw cycles. % Recovery is calculated by comparing the value of the treated sample to the freshly thawed control sample multiplied by 100. For some analytes, Plasma 3 and Serum 3 were spiked with recombinant standard. Samples reported as <LOW> are below the LLOQ. Antigen stability was determined by leaving samples at room temperature and 4°C for the times listed below. % Recovery is calculated by comparing the value of the treated sample to the freshly thawed control sample multiplied by 100. For some analytes, Plasma 3 and Serum 3 were spiked with recombinant standard.

### Statistical Methods

All analyses were performed using R version 2.12 or higher. Analytes that had a high number of missing values (e.g. greater than 40% of the samples had values below limits of detection) were excluded from the analyses. P-values graphically depicted are indicated by asterisks or plus signs in the following manner: p<0.001 by ***, p<0.01 by ** and p<0.05 by *.

In the discovery phase, candidate biomarkers were identified based on their association with Hammersmith score and other clinical outcomes in both univariate and multivariate analyses including ANOVA, t-test and Pearson correlation and by multivariate regression analysis (linear, lasso, random forest). Default values of the R functions were used for model tuning parameters (such as lambda for lasso and mtry for random forest). These analyses examined biomarker associations with multiple dependent variables: MHFMS scores, SMN2 copy number, SMN protein levels, and quantity of SMN2 full length, SMN-full length, SMN delta7, and total SMN transcripts. Analytes with significant association with one or more clinical variables in the discovery phase were candidates for inclusion in the validation phase, subject to non-statistical criteria such as assay performance, known biological relationships, and frequency of values at or near the lower limits of quantification or detection.

Univariate analysis in the validation phase identified 13 analytes as the best predictors. Because motor scale values are censored at 0 and 40, we implemented Tobit regression models to predict motor function scores [27]. We examined subsets of the 13 using best subsets analysis. All 13 analytes were entered as candidates in the models. Performance of the models was compared using adjusted R^2^ values calculated on bootstrap (out-of-bag) samples. Two Tobit models are reported; one using the 13 selected analytes (data not shown) and one using the 6 analytes in the best subset resulting from the best subsets analysis. MHFMS scores are bounded by floor (0) and ceiling (40) values, so we also examined Tobit models excluding these extremes. Excluding 0 and 40 MHFMS scores reduced the analytes’ predictive power, so this was not pursued.

## Supporting Information

Table S1 **iTRAQ workflow for BforSMA samples.** Samples tested in 8-plex format iTRAQ were run in randomized sets of 6 individually tagged samples alongside 2 reference standards consisting of pooled mixtures of all BforSMA samples. IVn refers to the set in which that each sample was tested. Type refers to whether the subject was an SMA patient with type status (1,2,3) or a Control. MHFMS = Modified Hammersmith Functional Motor Scale.
(DOCX)

Table S2 **Top 35 and other analytes in the pilot MAP.** The top 35 analytes selected via multivariate modeling for testing with the PNCRN NHS samples are listed with details on which analysis they were identified as hits (origin), whether they are motor regressors (MHFMS+), whether they regress to SMN2 copy number (#), SMN protein levels, quantity of SMN2 full length transcript, SMN-full length transcript, SMN delta7 transcript, or total SMN transcripts. The other 91 analytes assessed are also listed.
(DOCX)

Table S3 **Validation results for the SMA-MAP panel.** Assays for CD93, ENG, ERBB2, and IGF1 had minor issues with cross-reactivity or dilutional linearity that are ameliorated with dilution modification still within assay dynamic ranges. CLEC3B measurements were imprecise when analyte levels were close to the lower limit of quantitation. CLEC3B spike recovery could be reduced due to the antibodies binding both monomeric and tetrameric forms in the matrix while using a monomeric assay standard. Plasma samples for CCL2, CLEC3B, and ERBB2 were unstable at room temperature for >4 hours. Matrix interference measures were conducted with spikes of up to 500mg/dL hemoglobin or triglyceride, or 20 mg/dL bilirubin. Dilutions tested were 1∶10, 1∶20, and 1∶40. Freeze thaw values shown are from the third freeze thaw cycle. Antigen stability range represents the signal present with sample storage for 2 h at 4°C to 24 h at room temperature. *Indicates that there was 20% interference when COMP is present with THBS4; the analytes are known to bind *in vivo*.
(DOCX)

Table S4 **Adjusted R^2^ of the top 13 analytes predicted SMA motor and non-motor outcomes to actual patient values using the PNCR NHS.** The adjusted R^2^ values were based on the linear regression to predicted outcome measures using the 13 motor analytes with and without age of onset as a clinical covariate. Predictive ability is similar among the motor scales, and correlation values are generally greater for the motor scales than the non-motor outcomes with the exception of pulmonary function (FVC).
(DOCX)
