# Supplementary material for: SMA-MAP: A Plasma Protein Panel for Spinal Muscular Atrophy
Source: PLoS One. 2013 Apr 2;8(4):e60113. doi: 10.1371/journal.pone.0060113 (PMC3615018; doi:10.1371/journal.pone.0060113)
Supplement: Table S1 — iTRAQ workflow for BforSMA samples. Samples tested in 8-plex format iTRAQ were run in randomized sets of 6 individually tagged samples alongside 2 reference standards consisting of pooled mixtures of all BforSMA samples. IVn refers to the set in which that each sample was tested. Type refers to whether the subject was an SMA patient with type status (1,2,3) or a Control. MHFMS = Modified Hammersmith Functional Motor Scale. (DOCX) [file pone.0060113.s001.docx]

**Table S1. iTRAQ workflow for BforSMA samples**

| Subject | Set | Type | Age (y) | Gender | MHFMS |
| --- | --- | --- | --- | --- | --- |
| A8800040140 | IV1 | 1 | 2.4 | M | 0 |
| A8800050140 | IV17 | 2 | 3.5 | M | 7 |
| A8800120140 | IV2 | 1 | 3.5 | F | 0 |
| A8800130140 | IV18 | 2 | 2.2 | M | 1 |
| A8800140140 | IV1 | Control | 3.3 | M | 40 |
| A8800150140 | IV19 | 2 | 7.6 | F | 10 |
| A8800160140 | IV2 | Control | 10.9 | M | 40 |
| A8800180140 | IV20 | 2 | 2.4 | M | 0 |
| A8800190140 | IV1 | 3 | 4.9 | F | 31 |
| A8800200140 | IV21 | 2 | 2.6 | F | 24 |
| A8800220140 | IV2 | 3 | 11.8 | M | 40 |
| A8800230140 | IV3 | 3 | 6.9 | F | 36 |
| A8800240140 | IV4 | 3 | 5.6 | F | 40 |
| A8800250140 | IV5 | 3 | 8.0 | M | 40 |
| A8800260140 | IV22 | 2 | 3.0 | F | 18 |
| A8800300140 | IV1 | 2 | 5.2 | M | 0 |
| A8800310140 | IV2 | 2 | 5.7 | M | 28 |
| A8800320140 | IV3 | 2 | 2.6 | F | 12 |
| A8800340140 | IV3 | Control | 5.9 | M | 40 |
| A8800350140 | IV4 | 2 | 13.0 | M | 0 |
| A8800360140 | IV5 | 2 | 10.0 | F | 12 |
| A8800370140 | IV6 | 2 | 2.8 | F | 36 |
| A8800380140 | IV3 | 1 | 5.0 | M | 0 |
| A8800390140 | IV7 | 2 | 9.4 | F | 2 |
| A8800400140 | IV6 | 3 | 9.7 | M | 38 |
| A8800410140 | IV7 | 3 | 9.2 | F | 39 |
| A8800440140 | IV8 | 2 | 4.5 | M | 10 |
| A8800460140 | IV9 | 2 | 8.4 | M | 13 |
| A8800490140 | IV23 | 2 | 11.1 | M | 2 |
| A8800510140 | IV24 | 2 | 3.2 | M | 25 |
| A8800530140 | IV4 | Control | 4.0 | M | 39 |
| A8800540140 | IV4 | 1 | 7.9 | M | 0 |
| A8800550140 | IV12 | 2 | 4.3 | M | 20 |
| A8800560140 | IV8 | 3 | 10.4 | F | 40 |
| A8800570140 | IV9 | 3 | 5.3 | M | 39 |
| A8800580140 | IV10 | 3 | 7.6 | F | 40 |
| A8800590140 | IV5 | Control | 5.2 | F | 40 |
| A8800600140 | IV13 | 2 | 10.4 | F | 14 |
| A8800610140 | IV24 | 3 | 11.1 | F | 19 |
| A8800620140 | IV14 | 2 | 8.0 | F | 10 |
| A8800630140 | IV15 | 2 | 9.4 | F | 2 |
| A8800650140 | IV6 | Control | 12.6 | M | 40 |
| A8800660140 | IV16 | 2 | 2.3 | M | 27 |
| A8800670140 | IV12 | 3 | 11.9 | F | 34 |
| A8800680140 | IV7 | Control | 3.5 | F | 40 |
| A8800690140 | IV8 | Control | 3.2 | F | 40 |
| A8800710140 | IV9 | Control | 9.5 | F | 40 |
| A8800720140 | IV17 | 2 | 2.6 | M | 10 |
| A8800730140 | IV23 | Control | 9.7 | F | 40 |
| A8800740140 | IV13 | Control | 4.8 | F | 40 |
| A8800770140 | IV5 | 1 | 12.7 | M | 0 |
| A8800790140 | IV14 | 3 | 7.0 | M | 33 |
| A8800810140 | IV15 | 3 | 6.9 | M | 40 |
| A8800820140 | IV18 | 2 | 5.1 | F | 9 |
| A8800830140 | IV16 | 3 | 9.8 | M | 40 |
| A8800850140 | IV24 | Control | 10.8 | M | 40 |
| A8800880140 | IV19 | 2 | 2.5 | M | 29 |
| A8800900140 | IV18 | 3 | 4.5 | F | 38 |
| A8800910140 | IV19 | 3 | 12.9 | M | 36 |
| A8800920140 | IV20 | 3 | 3.8 | M | 37 |
| A8800940140 | IV21 | 3 | 4.4 | M | 40 |
| A8800950140 | IV20 | 2 | 5.5 | F | 17 |
| A8800960140 | IV12 | Control | 7.4 | F | 40 |
| A8800970140 | IV13 | Control | 10.3 | F | 40 |
| A8800980140 | IV1 | 3 | 7.9 | F | 40 |
| A8801020140 | IV21 | 2 | 6.5 | F | 19 |
| A8801030140 | IV14 | Control | 5.0 | M | 40 |
| A8801040140 | IV15 | 3 | 12.1 | M | 40 |
| A8801050140 | IV16 | Control | 7.9 | F | 40 |
| A8801060140 | IV2 | 3 | 9.5 | F | 40 |
| A8801080140 | IV17 | Control | 5.7 | F | 40 |
| A8801090140 | IV18 | Control | 10.9 | M | 40 |
| A8801100140 | IV6 | 1 | 11.9 | M | 0 |
| A8801130140 | IV7 | 3 | 10.0 | M | 18 |
| A8801140140 | IV3 | 3 | 10.0 | M | 19 |
| A8801150140 | IV19 | Control | 2.6 | M | 40 |
| A8801180140 | IV22 | 2 | 13.0 | F | 4 |
| A8801200140 | IV1 | 2 | 12.4 | F | 4 |
| A8801220140 | IV2 | 2 | 10.7 | F | 13 |
| A8801230140 | IV4 | 3 | 7.3 | F | 22 |
| A8801240140 | IV3 | 2 | 6.7 | M | 9 |
| A8801270140 | IV6 | 3 | 5.5 | F | 40 |
| A8801300140 | IV4 | 2 | 10.6 | M | 32 |
| A8801310140 | IV7 | 3 | 2.8 | M | 38 |
| A8801330140 | IV5 | 2 | 3.6 | M | 30 |
| A8801340140 | IV20 | Control | 11.4 | F | 40 |
| A8801350140 | IV21 | Control | 6.1 | M | 40 |
| A8801360140 | IV8 | 3 | 10.6 | F | 2 |
| A8801380140 | IV9 | 3 | 2.6 | M | 26 |
| A8801390140 | IV6 | 2 | 7.5 | M | 10 |
| A8801400140 | IV7 | 2 | 10.1 | F | 25 |
| A8801410140 | IV23 | 3 | 4.9 | M | 40 |
| A8801430140 | IV8 | 2 | 5.4 | M | 10 |
| A8801440140 | IV8 | 1 | 3.0 | F | 0 |
| A8801480140 | IV9 | 1 | 4.6 | M | 0 |
| A8801490140 | IV22 | Control | 2.2 | F | 37 |
| A8801530140 | IV11 | 1 | 3.6 | F | 0 |
| A8801640140 | IV9 | 2 | 11.1 | M | 13 |
| A8801650140 | IV23 | 2 | 9.3 | F | 34 |
| A8801670140 | IV24 | 2 | 7.0 | M | 8 |
| A8801680140 | IV12 | 2 | 2.4 | M | 11 |
| A8801690140 | IV23 | 1 | 4.0 | M | 0 |
| A8801710140 | IV12 | 3 | 8.9 | M | 40 |
| A8801720140 | IV13 | 2 | 3.1 | F | 30 |
| A8801740140 | IV13 | 3 | 10.1 | F | 40 |
| A8801750140 | IV14 | 3 | 2.4 | F | 27 |
| A8801760140 | IV14 | 2 | 7.5 | M | 28 |
| A8801770140 | IV15 | 3 | 12.7 | F | 8 |
| A8801780140 | IV16 | 3 | 3.2 | F | 40 |
| A8801800140 | IV17 | 3 | 3.7 | F | 40 |
| A8801850140 | IV18 | 3 | 6.1 | F | 40 |
| A8801950140 | IV11 | 1 | 6.6 | M | 0 |
| A8801960140 | IV12 | 1 | 7.7 | F | 0 |
| A8801980140 | IV15 | 1 | 3.8 | M | 0 |
| A8802450140 | IV16 | 2 | 3.1 | F | 1 |
| A8802470140 | IV13 | 2 | 8.7 | M | 8 |
| A8802480140 | IV19 | 1 | 3.0 | M | 0 |
| A8802500140 | IV17 | 3 | 11.2 | M | 40 |
| A8802540140 | IV20 | 2 | 2.3 | M | 0 |
| A8802940140 | IV24 | 3 | 3.2 | F | 11 |
| A8802960140 | IV14 | 2 | 9.6 | M | 31 |
| A8803070140 | IV19 | 1 | 12.2 | F | 0 |
| A8803110140 | IV20 | 2 | 4.4 | F | 11 |
| A8803130140 | IV21 | 2 | 11.3 | F | 11 |
| A8803180140 | IV21 | 3 | 4.1 | M | 40 |
| A8803310140 | IV15 | 2 | 9.1 | F | 8 |
| A8803360140 | IV16 | 1 | 2.5 | F | 0 |
