# Supplementary material for: SMA-MAP: A Plasma Protein Panel for Spinal Muscular Atrophy
Source: PLoS One. 2013 Apr 2;8(4):e60113. doi: 10.1371/journal.pone.0060113 (PMC3615018; doi:10.1371/journal.pone.0060113)
Supplement: Table S2 — Top 35 and other analytes in the pilot MAP. The top 35 analytes selected via multivariate modeling for testing with the PNCRN NHS samples are listed with details on which analysis they were identified as hits (origin), whether they are motor regressors (MHFMS+), whether they regress to SMN2 copy number (#), SMN protein levels, quantity of SMN2 full length transcript, SMN-full length transcript, SMN delta7 transcript, or total SMN transcripts. The other 91 analytes assessed are also listed. (DOCX) [file pone.0060113.s002.docx]

**Table S2. Top 35 and other analytes in the pilot MAP**

|  |  |  | Rationale |  |  | Transcript |  |  |  |
| --- | --- | --- | --- | --- | --- | --- | --- | --- | --- |
|  | Protein | Origin | MHFMS+ | SMN2 # | SMN protein | SMN2-fl | SMN-fl | SMN-d7 | SMN-total |
| Top 35 Markers | ANGPT2 | MAP | Yes |  |  |  |  | Yes |  |
|  | APCS | LC/MS+MAP |  | Yes |  |  |  |  |  |
|  | APOA4 | LC/MS+MAP | Yes |  |  |  | Yes |  |  |
|  | ASHG | MAP |  |  |  | Yes |  |  |  |
|  | AXL | MAP | Yes |  | Yes |  |  |  |  |
|  | CCL16 | MAP | Yes |  |  |  |  |  |  |
|  | CCL2 | MAP | Yes |  |  |  |  |  |  |
|  | CCL22 | MAP | Yes |  |  |  |  |  |  |
|  | CD93 | LC/MS |  |  |  |  |  |  |  |
|  | CDH13 | LC/MS |  |  |  |  |  |  |  |
|  | CFH | MAP | Yes |  |  |  |  |  |  |
|  | CHI3L1 | MAP | Yes |  |  |  |  |  |  |
|  | CLEC3B | LC/MS+MAP | Yes | Yes | Yes |  |  |  |  |
|  | COMP | LC/MS | Yes | Yes |  |  |  |  |  |
|  | CRP | LC/MS+MAP |  | Yes |  |  |  |  |  |
|  | CTSD | LC/MS+MAP | Yes |  |  |  |  | Yes |  |
|  | DPP4 | LC/MS | Yes |  |  |  |  |  |  |
|  | ENG | LC/MS+MAP | Yes | Yes | Yes |  |  |  |  |
|  | ERBB2 | MAP | Yes |  | Yes |  |  |  |  |
|  | ERBB3 | MAP |  | Yes |  |  |  |  |  |
|  | FBLN1 | MAP | Yes |  |  |  |  |  |  |
|  | GLO1 | MAP | Yes |  |  |  |  |  |  |
|  | IGFBP6 | LC/MS+MAP | Yes |  |  |  | Yes |  | Yes |
|  | KLK5 | MAP | Yes |  |  |  |  |  |  |
|  | LEP | MAP | Yes | Yes | Yes |  |  |  |  |
|  | LUM | LC/MS |  |  |  |  |  |  |  |
|  | MB | MAP | Yes |  |  |  |  |  |  |
|  | MMP7 | MAP | Yes |  |  |  |  |  |  |
|  | PEPD | LC/MS |  |  |  |  |  |  |  |
|  | PGF | MAP | Yes |  |  |  | Yes |  | Yes |
|  | PPY | MAP | Yes |  |  | Yes |  | Yes |  |
|  | SPP1 | MAP | Yes | Yes |  |  |  |  |  |
|  | THBS4 | LC/MS |  |  |  |  |  |  |  |
|  | TNFR1 | MAP | Yes |  |  |  | Yes |  | Yes |
|  | TNXB | LC/MS | Yes | Yes |  |  |  |  |  |
| Additional analytes | A2M | MAP | Yes |  |  |  |  |  |  |
|  | ACE | MAP | Yes |  | Yes |  |  |  |  |
|  | ADIPOQ | MAP |  |  |  |  |  |  |  |
|  | ANG | MAP |  |  |  |  |  |  |  |
|  | APOB | MAP |  |  |  |  |  |  |  |
|  | AST | MAP |  |  |  |  |  |  |  |
|  | B2M | MAP | Yes |  |  |  |  |  |  |
|  | BCL2L2 | MAP |  |  |  |  |  |  |  |
|  | BDNF | MAP |  |  |  |  |  |  |  |
|  | CA 15-3 | MAP |  |  |  |  |  |  |  |
|  | CA 72-4 | MAP |  |  |  |  |  |  |  |
|  | CCL24 | MAP | Yes |  |  |  |  |  |  |
|  | CCL26 | MAP |  |  |  |  |  |  |  |
|  | CCL4 | MAP |  |  |  |  |  |  |  |
|  | CCL5 | MAP |  |  | Yes |  | Yes |  |  |
|  | CD5 | MAP |  |  |  |  |  |  |  |
|  | CSF1 | MAP | Yes |  | Yes |  |  |  |  |
|  | CSF2 | MAP |  |  |  |  |  |  |  |
|  | CXCL13 | MAP |  |  |  |  |  |  |  |
|  | CXCL5 | MAP |  |  |  |  |  |  |  |
|  | EZR | MAP |  |  |  |  |  |  |  |
|  | FABP4 | MAP | Yes |  |  |  |  |  |  |
|  | FASLGR | MAP |  |  |  |  |  |  |  |
|  | FLT1 | MAP | Yes |  |  |  |  |  |  |
|  | FRTN | MAP |  |  |  |  |  |  |  |
|  | FSH | MAP |  |  |  |  | Yes |  |  |
|  | G6PI | MAP |  |  |  | Yes |  |  |  |
|  | GLP1 | MAP |  |  |  |  |  |  |  |
|  | GSN | LC/MS+MAP |  |  |  |  |  |  |  |
|  | HGF | MAP |  |  |  |  |  |  |  |
|  | HSP60 | MAP |  |  |  |  |  |  |  |
|  | IGFBP1 | MAP |  |  |  |  |  |  |  |
|  | IGFBP2 | MAP |  |  |  |  |  |  |  |
|  | IGFBP3 | MAP |  |  |  |  |  |  |  |
|  | IGFBP4 | MAP |  |  |  |  |  |  |  |
|  | IGFBP5 | LC/MS |  |  |  |  |  |  |  |
|  | IL10 | MAP |  |  |  |  |  | Yes |  |
|  | IL12P40 | MAP |  |  | Yes |  |  |  |  |
|  | IL12P70 | MAP |  |  |  |  |  |  |  |
|  | IL13 | MAP |  |  |  |  |  |  |  |
|  | IL15 | MAP |  |  |  |  |  |  |  |
|  | IL16 | MAP |  |  |  |  |  |  |  |
|  | IL18 | MAP |  |  |  |  | Yes |  |  |
|  | IL1A | MAP | Yes |  |  |  |  |  |  |
|  | IL2 | MAP |  |  |  |  |  |  |  |
|  | IL2RA | MAP |  |  |  |  |  |  |  |
|  | IL3 | MAP |  |  | Yes |  |  |  |  |
|  | IL4 | MAP |  |  |  |  |  |  |  |
|  | IL5 | MAP |  |  |  |  |  |  |  |
|  | IL6RB | MAP | Yes |  |  |  |  |  |  |
|  | IL7 | MAP |  |  | Yes |  |  |  |  |
|  | IL8 | MAP | Yes |  |  |  |  |  |  |
|  | INS | MAP |  |  |  |  |  |  |  |
|  | LGALS3 | MAP |  |  |  |  |  |  |  |
|  | LH | MAP |  |  |  |  |  |  |  |
|  | LPA | MAP |  |  |  |  |  |  |  |
|  | MICA | MAP |  |  |  |  |  |  |  |
|  | MIF | MAP | Yes |  |  |  |  |  |  |
|  | MMP1 | MAP |  |  |  |  |  |  |  |
|  | MMP10 | MAP |  |  |  |  |  |  |  |
|  | MMP2 | MAP | Yes |  | Yes |  |  | Yes |  |
|  | MMP9 | MAP | Yes |  |  |  |  |  |  |
|  | MSP | MAP | Yes |  |  |  |  |  |  |
|  | OPG | MAP |  |  |  |  |  |  |  |
|  | PAI1 | MAP | Yes |  |  |  |  |  |  |
|  | PARC | MAP |  |  |  |  |  |  |  |
|  | PGI | MAP |  |  |  |  |  |  |  |
|  | PRL | MAP |  |  |  |  |  |  |  |
|  | Pro-INS intact | MAP |  |  |  |  | Yes |  |  |
|  | Pro-INS total | MAP |  |  |  |  |  |  | Yes |
|  | RETN | MAP |  |  |  |  |  |  |  |
|  | S100A4 | MAP | Yes |  |  |  |  |  | Yes |
|  | SCF | MAP |  |  | Yes |  |  |  |  |
|  | SERPINEA3 | MAP |  |  |  |  |  |  |  |
|  | SHBG | LC/MS |  |  |  |  |  |  |  |
|  | TBG | MAP |  |  |  |  |  |  |  |
|  | TG | MAP |  |  |  |  |  |  |  |
|  | TGFB1 | MAP | Yes |  |  |  |  |  |  |
|  | THPO | MAP |  |  | Yes |  |  |  |  |
|  | TIE2 | MAP |  |  |  |  |  |  | Yes |
|  | TIMP1 | MAP |  |  | Yes |  |  |  |  |
|  | TNFB | MAP |  |  |  |  |  |  |  |
|  | TNFR2 | MAP |  |  |  |  |  |  |  |
|  | TPA | MAP |  |  |  |  |  |  |  |
|  | TRAILR3 | MAP |  |  |  |  |  | Yes |  |
|  | UPA | MAP |  |  |  |  |  | Yes |  |
|  | VCAM1 | LC/MS+MAP | Yes |  | Yes |  |  |  |  |
|  | VKDPS | MAP | Yes |  |  |  |  |  |  |
|  | VTN | LC/MS+MAP | Yes |  |  |  |  |  |  |
|  | VWF | MAP |  |  |  |  |  |  |  |
|  | XCL1 | MAP |  |  |  |  |  |  |  |
