# Supplementary material for: SMA-MAP: A Plasma Protein Panel for Spinal Muscular Atrophy
Source: PLoS One. 2013 Apr 2;8(4):e60113. doi: 10.1371/journal.pone.0060113 (PMC3615018; doi:10.1371/journal.pone.0060113)
Supplement: Table S3 — Validation results for the SMA-MAP panel. Assays for CD93, ENG, ERBB2, and IGF1 had minor issues with cross-reactivity or dilutional linearity that are ameliorated with dilution modification still within assay dynamic ranges. CLEC3B measurements were imprecise when analyte levels were close to the lower limit of quantitation. CLEC3B spike recovery could be reduced due to the antibodies binding both monomeric and tetrameric forms in the matrix while using a monomeric assay standard. Plasma samples for CCL2, CLEC3B, and ERBB2 were unstable at room temperature for >4 hours. Matrix interference measures were conducted with spikes of up to 500mg/dL hemoglobin or triglyceride, or 20 mg/dL bilirubin. Dilutions tested were 1∶10, 1∶20, and 1∶40. Freeze thaw values shown are from the third freeze thaw cycle. Antigen stability range represents the signal present with sample storage for 2 h at 4°C to 24 h at room temperature. *Indicates that there was 20% interference when COMP is present with THBS4; the analytes are known to bind in vivo. (DOCX) [file pone.0060113.s003.docx]

**Table S3. Validation results for the SMA-MAP panel**

| Analyte | Units | LDD | LLOQ | Dynamic Range | Cross-reactivity | CV% | Linearity | Spike | Matrix Interference | Freeze-thaw | Antigen stability |
| --- | --- | --- | --- | --- | --- | --- | --- | --- | --- | --- | --- |
| APOB | ug/L | 2.5 | 11 | 2.5-12420 | <1% | <10% | 122% | 78% | 88, 120, 73% | 125% | 101-88% |
| AXL | ng/mL | 0.055 | 0.12 | 0.055-276 | <1% | <15% | 51% | 76% | 100, 96, 109% | 102% | 93-79% |
| CHD13 | ng/mL | 1.3 | 1.8 | 0.25-1272 | <1% | <10% | 117% | 105% | 97, 99, 106% | 99% | 90-84% |
| COMP | ng/mL | 4.5 | 8.4 | 4.5-22600 | <1%* | <10% | 85% | 85% | 98, 94, 98% | 108% | 103-104% |
| CTSD | ng/mL | 35 | 33 | 10-50000 | <1% | <15% | 121% | 97% | 90, 127, 85% | 93% | 90-87% |
| CFH | ug/mL | 38 | 43 | 5.2-25800 | <1% | <10% | 104% | 92% | 95, 113, 105% | 80% | 109-89% |
| CD93 | ug/mL | 0.014 | 0.02 | 0.014-70 | <1% | <10% | 93% | 83% | 104, 99, 97% | 106% | 104-95% |
| CRP | ug/mL | 0.0091 | 0.0066 | 0.0057-28 | <1% | <10% | 94% | 89% | 108, 117, 113% | 96% | 119-109% |
| DPP4 | ng/mL | 12 | 31 | 7.9-39400 | <1% | <10% | 100% | 96% | 93, 92 , 92% | 112% | 97% |
| ENG | ng/mL | 0.0084 | 0.012 | .0084-42 | <1% | <10% | 62% | 121% | 98, 98, 108% | 102% | 94-83% |
| ERBB2 | ng/mL | 0.015 | 0.03 | .01-52 | <1% | <10% | 68% | 112% | 106, 99, 108% | 91% | 85-62% |
| ASHG | ug/mL | 3.6 | 8.1 | 3.6-18260 | <1% | <10% | 116% | 97% | 95, 109, 104% | 84% | 118-103% |
| FBLN1 | ug/mL | 0.061 | 0.069 | 0.04-200 | <1% | <10% | 99% | 90% | 88, 113, 87% | 101% | 91-85% |
| IGF1 | ng/mL | 10 | 14 | 3.6-18200 | <1% | <10% | 69% | 102% | 104, 93, 102% | 102% | 96-77% |
| IGFBP6 | ng/mL | 2.5 | 3.2 | 0.36-1821 | <1% | <15% | 95% | 71% | 102, 99, 102% | 101% | 94-75% |
| LEP | ng/mL | 0.18 | 0.12 | 0.21-105 | <1% | <10% | 97% | 97% | 108, 98, 108% | 95% | 92-81% |
| LUM | ug/mL | 0.7 | 0.96 | 0.18-928 | <1% | <10% | 123% | 163% | 100, 94, 89% | 104% | 116-105% |
| CCL2 | ng/mL | 16 | 18 | 3.6-18116 | <1% | <10% | 86% | 90% | 111,90, 98% | 95% | 89-69% |
| MB | ng/mL | 0.97 | 1.4 | 0.46-2340 | <1% | <15% | 95% | 84% | 81, 99, 121% | 112% | 97-101% |
| SPP1 | ng/mL | 1.2 | 1.6 | 0.15-754 | <1% | <10% | 72% | 89% | 104, 100, 92% | 93% | 94-77% |
| PEPD | ug/mL | 0.35 | 0.24 | 0.092-464 | <1% | <10% | 103% | 85% | 96, 91, 93% | 108% | 103-91% |
| PGF | ng/mL | 7.1 | 20 | 7.1-35649 | <1% | <10% | 97% | 97% | 106, 102, 114% | 94% | 90-74% |
| ASCS | ug/mL | 0.16 | 0.23 | 0.037-185 | <1% | <20% | 97% | 93% | 96, 94, 96% | 102% | 115-102% |
| TNXB | ng/mL | 3.0 | 4.3 | 1.4-6925 | <1% | <10% | 104% | 94% | 112, 102, 111% | 96% | 93-78% |
| CLEC3B | ug/mL | 0.81 | 2.6 | 0.5-2500 | <1% | <20% | 89% | 47% | 116, 81, 96% | 96% | 87-65% |
| THBS4 | ug/mL | 0.14 | 0.19 | 0.10-506 | <1%* | <10% | 105% | 97% | 92, 92, 97% | 117% | 100-112% |
| CHI3L1 | ng/mL | 4.0 | 5.8 | 4.0-20000 | <1% | <10% | 96% | 98% | 88, 72, 91% | 99% | 98-93% |
