# Supplementary material for: SMA-MAP: A Plasma Protein Panel for Spinal Muscular Atrophy
Source: PLoS One. 2013 Apr 2;8(4):e60113. doi: 10.1371/journal.pone.0060113 (PMC3615018; doi:10.1371/journal.pone.0060113)
Supplement: Table S4 — Adjusted R2 of the top 13 analytes predicted SMA motor and non-motor outcomes to actual patient values using the PNCR NHS. The adjusted R2 values were based on the linear regression to predicted outcome measures using the 13 motor analytes with and without age of onset as a clinical covariate. Predictive ability is similar among the motor scales, and correlation values are generally greater for the motor scales than the non-motor outcomes with the exception of pulmonary function (FVC). (DOCX) [file pone.0060113.s004.docx]

**Table S4. Adjusted R^2^ of the top 13 analytes predicted SMA motor and non-motor outcomes to actual patient values using the PNCR NHS**

|  | |  | |  |  | |  |  |  |
| --- | --- | --- | --- | --- | --- | --- | --- | --- | --- |
|  | | No covariate | | Age of onset Covariate | | |  |  |  |
| HFMS | | 0.37 | | 0.51 | | |  |  |  |
| HFMSE | | 0.33 | | 0.56 | | |  |  |  |
| GMFM88 | | 0.29 | | 0.53 | | |  |  |  |
| HighestMF | | 0.12 | | 0.29 | | |  |  |  |
| FVC | | 0.33 | | 0.62 | | |  |  |  |
| FVC% | | 0.45 | | 0.54 | | |  |  |  |
| MyoEF | | 0.10 | | 0.43 | | |  |  |  |
| MyoKE | | 0.24 | | 0.25 | | |  |  |  |
| MyoKF | | -0.003 | | 0.04 | | |  |  |  |
| CMAP | | -0.06 | | 0.24 | | |  |  |  |
| MUNE | | -0.06 | | 0.25 | | |  |  |  |
